# Structural and functional features of a broad-spectrum prophage-encoded enzybiotic from *Enterococcus faecium*

**DOI:** 10.1038/s41598-023-34309-2

**Published:** 2023-05-08

**Authors:** Georgios E. Premetis, Angeliki Stathi, Anastassios C. Papageorgiou, Nikolaos E. Labrou

**Affiliations:** 1 Laboratory of Enzyme Technology, Department of Biotechnology, School of Applied Biology and Biotechnology, Agricultural University of Athens, 75 Iera Odos Street, 11855 Athens, Greece; 2 Department of Microbiology, “Aghia Sophia” Children’s Hospital, 11527 Athens, Greece; 3 Turku Bioscience Centre, University of Turku and Åbo Akademi University, 20521 Turku, Finland

**Keywords:** Biochemistry, Biophysical chemistry, Enzymes

## Abstract

Multidrug-resistant (MDR) bacteria have become a growing threat to public health. The gram-positive *Enterococcus faecium* is classified by WHO as a high-priority pathogen among the global priority list of antibiotic-resistant bacteria. Peptidoglycan-degrading enzymes (PDEs), also known as enzybiotics, are useful bactericidal agents in the fight against resistant bacteria. In this work, a genome-based screening approach of the genome of *E. faecium* allowed the identification of a putative PDE gene with predictive amidase activity (*Ef*Ami1; EC 3.5.1.28) in a prophage-integrated sequence. *Ef*Ami1 is composed by two domains: a N-terminal Zn^2+^-dependent *N*-acetylmuramoyl-l-alanine amidase-2 (NALAA-2) domain and a C-terminal domain with unknown structure and function. The full-length gene of *Ef*Ami1 was cloned and expressed as a 6xHis-tagged protein in *E. coli*. *Ef*Ami1 was produced as a soluble protein, purified, and its lytic and antimicrobial activities were investigated using turbidity reduction and Kirby–Bauer disk-diffusion assays against clinically isolated bacterial pathogens. The crystal structure of the N-terminal amidase-2 domain was determined using X-ray crystallography at 1.97 Å resolution. It adopts a globular fold with several α-helices surrounding a central five-stranded β-sheet. Sequence comparison revealed a cluster of conserved amino acids that defines a putative binding site for a buried zinc ion. The results of the present study suggest that *Ef*Ami1 displays high lytic and antimicrobial activity and may represent a promising new antimicrobial in the post-antibiotic era.

## Introduction

Over the past century, the discovery of new antibiotics and the effectiveness of old ones have been continuously declining, suggesting that antimicrobial resistance (AMR) has important health and economic dangers both at the individual and population levels^1,2^. The World Health Organization (WHO) considers that the ESKAPE family of pathogens (*Escherichia coli, Staphylococcus aureus, Klebsiella pneumoniae, Acinetobacter baumannii, Pseudomonas aeruginosa, Enterococcus faecium*) is the leading cause of hospital-acquired infections worldwide^3^.

*Enterococcus faecium* is a gram-positive, aerobic bacterium found in a variety of environments, such as soil, water, and the intestinal tract of humans and other animals^4^. These bacteria were considered safe without causing significant infections but, after the 1980s, they evolved as severe nosocomial pathogens^5–8^. Their ability to survive for long periods of time on hospital surfaces (benches, beds, surgical devices, and ventilation systems) poses an increasing difficulty in controlling their spread^9^. Antimicrobial resistant enterococci are now a major cause of hospital-acquired infections mainly of bloodstream and urinary tract^5,10–12^. *Enterococcus faecium* is a growing cause of associated urinary tract infections accounting for 15% of all cases in US, making it the second most common pathogen^13,14^.

The use of cell-wall lytic enzymes (so-called enzybiotics) to fight bacteria has become a viable alternative approach to cope with the crisis of antimicrobial resistance^15–19^. Compared with conventional antibiotics, enzybiotics display several advantages, such as rapid function, selectivity against microbial hosts, low chances of developing resistance, and high efficacy against multidrug-resistant bacteria^15,20^.

Cell-wall lytic enzymes are classified into two large groups: endolysins and autolysins. Endolysins are phage-encoded enzymes that have evolved to hydrolyze the cell wall of bacteria^21^. Autolysins hydrolyze specific bonds in the peptidoglycan backbone of the bacterial cell wall and play important roles in the control of cell growth, cell lysis, daughter-cell separation, and biofilm formation^22^. Cell wall lytic enzymes, based on their specificity and catalytic mechanisms on the bacterial peptidoglycan, are classified into three main groups: amidases, peptidases, and glycosidases^23,24^. Amidases are *N*-acetylmuramoyl-l-alanine amidases (NALAAs) that catalyze the hydrolysis of the amide bond between *N*-acetylmuramoyl residues and l-amino acid residues in certain bacterial cell-wall glycopeptides^24,25^. In bacteria, three different types of catalytic domains are currently reported as responsible for a NALAA activity^24^: (1) Type 2 (NALΑA-2, InterPro: IPR002502), (2) Type 3 (NALΑA-3, InterPro: IPR002508), and (3) Type 5 (NALΑA-5, InterPro: IPR008044).

Prophages (temperate phages) are phage genomes integrated into the host genome. They have the ability to enter the host cell and integrate into the host genome (lysogenic cycle), residing in the host cells as prophages until they are induced to start a lytic cycle^26,27^. Prophages represent an interesting source of new genes, and their genes/proteins are often associated with new functional features. Prophages, therefore, have attracted significant attention in recent years from a biotechnology point of view^26,27^. Prophage sequences of *Enterococcus faecium* genomes have recently been characterized^28,29^.

In the present study, a putative cell-wall lytic enzyme (*Ef*Ami1) with predictive amidase activity was identified in the prophage sequence integrated into the *Enterococcus faecium* 11C6_DIV0699 genome and characterized. The bacteriolytic activity of *Ef*Ami1 was assessed against a clinically isolated *Enterococcus faecium* strain as well as other ESKAPE bacterial pathogens. Furthermore, the crystal structure of *Ef*Ami1 was determined and the key amino acid residues involved in substrate binding or catalysis were predicted and discussed.

## Results and discussion

### Identification and in silico characterization of *Ef*Ami1

The genome of *Enterococus faecium* 11C6_DIV0699 (accession no. ASM214031v1) was BLASTp searched using as a query the sequence of ORF9 (GenBank accession no. AP009390) that encodes for an endolysin in the *E. faecalis* bacteriophage φEF24C. The sequence of a putative NALAA (NCBI accession number: WP_086274872.1) integrated into the prophage genome of *E. faecium* was selected for further analysis. Lytic enzymes, which belong to the zinc amidase family (EC 3.5.1.28) and display NALAA activity, have attracted significant attention because of their enhanced antimicrobial activity^30^. Prophages are a less investigated source of new functional endolysin genes and therefore hold significant potential for further exploitation^26,27^. Prophage sequences in *E. faecium* genomes have been recently characterised^28,29^. The gene of *Ef*Ami1 consists of 975-bp and encodes for a protein of 324 amino acids with predicted molecular mass 36,290 Da and isoelectric point 6.31.

The presence of conserved protein domains in the *Ef*Ami1 sequence was assessed following a search against InterPro^31^. *Ef*Ami1 comprises two autonomous domains: an N-terminal catalytic domain (amino acids 6–144) and an unclassified C-terminal domain (amino acids 186–324). The N-terminal domain is classified in the 002502 InterPro superfamily, which contains as a member the amidase-2 domain (smart00644). This domain belongs to the peptidoglycan recognition protein superfamily (PGRPs, cl02712). The amidase-2 family includes Zn^2+^-dependent NALAAs that cleave the amide bond between *N*-acetylmuramic acid and L-Ala in bacterial cell walls^24^.

The C-terminal domain lacks homology with any functional protein domain in InterPro. To elucidate the functional role of the C-terminal domain, the AlphaFold predicted model^32^ was inspected (https://www.alphafold.ebi.ac.uk/entry/A0A7V7GKT0). Based on AlphaFold prediction, the C-terminal domain of *Ef*Ami1 adopts a β-barrel fold, consisting of an eight-stranded β-barrel with three α-helices positioned around it (Supplementary Fig. 1). Structural comparison of the C-terminal domain with structures deposited in the PDB was performed using the DALI server^33^. The highest similarity was found with the carbohydrate-binding module of a fungal β-mannosidase (PDB id 4UOJ)^34^ (Supplementary Fig. 1). Furthermore, some similarity was also observed with the structure adopted by the Glycosyl Hydrolase family 25 enzymes (GHF-25). GHF-25 enzymes form an irregular β-barrel conformation consisting of eight β-strands surrounded by six α-helices^30^. The putative biological function of the C-terminal domain was also evaluated using the I-TASSER server and employing the COFACTOR^35^ and COACH^36^ programs to structure-based function annotation. The results of this analysis provide further hints that the C-terminal domain possesses carbohydrate-binding properties. It is therefore conceivable to assume that the C-terminal domain of *Ef*Ami1 probably represents a new carbohydrate-binding module for peptidoglycan recognition, relevant to that reported for other endolysins^24^.

**Figure 1:**
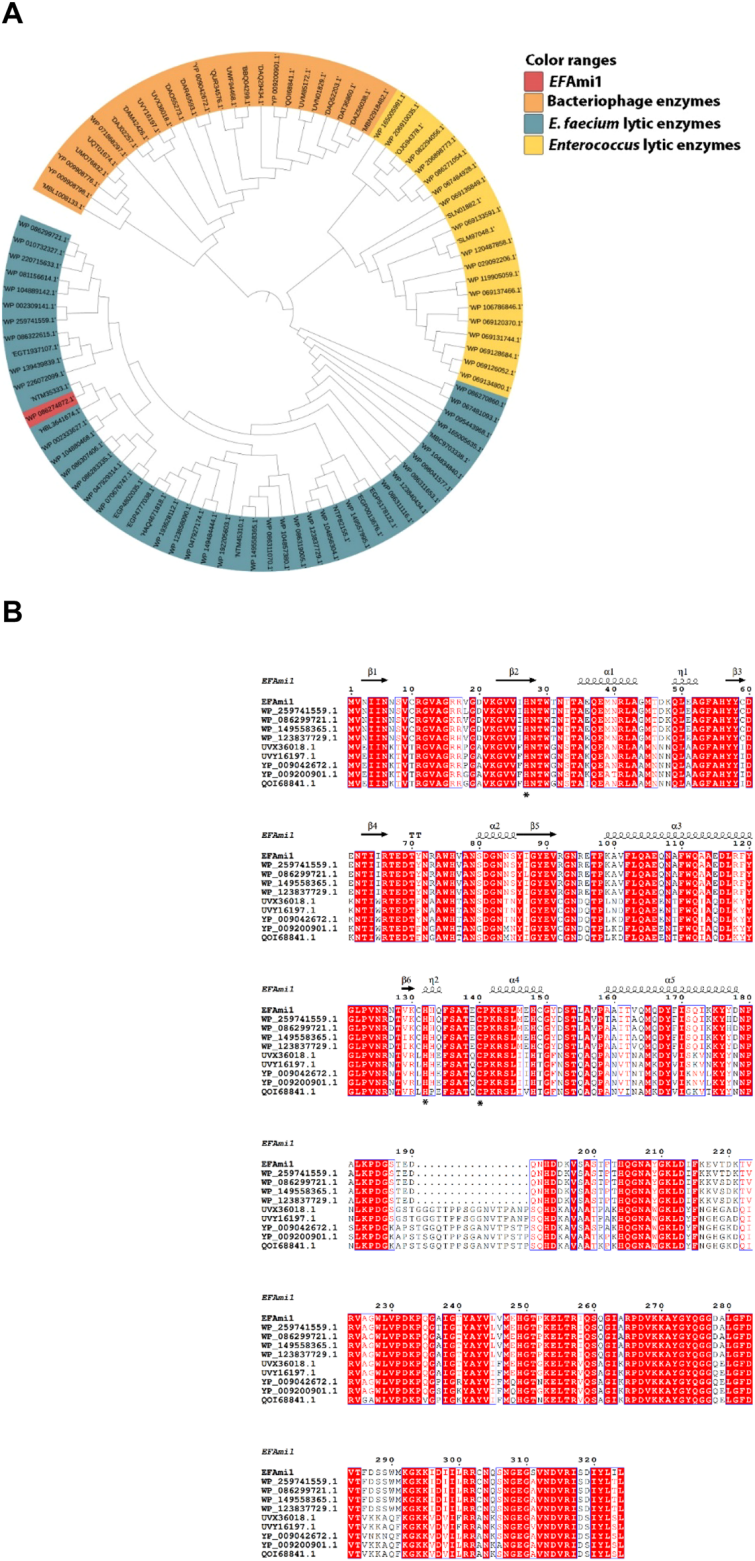
(**A**) Phylogenetic analysis of *Ef*Ami1. Multiple sequence alignment was achieved using Cobalt^37^. The phylogenetic tree was constructed with Phylip^38^ and displayed with iTOL v4^39^. (**B**) Multiple sequence alignment of NALAA homologous sequences with *Ef*Ami1. Alignment was performed using ClustalΟ^40^ and displayed using ESPript 3.0^41^. The NALAA sequences used (NCBI Accession number) were the following: WP_086274872.1 (*Ef*Ami1); WP_259741559.1 (from *Enterococcus faecium*); WP_086299721.1 (from *Enterococcus faecium*); WP_149558365.1 (from *Enterococcus faecium*); WP_123837729.1 (from *Enterococcus faecium*]); UVX36018.1 (from Bacteriophage sp); UVY16197.1 (from Bacteriophage sp); YP_009042672.1 (from *Enterococcus* phage IME-EFm1); YP_009200901.1 (from *Enterococcus* phage IME-EFm5); QOI68841.1 (from *Enterococcus* phage 9184). The zinc-binding residues are shown with stars. *Ef*Ami1 numbering is shown above the alignment. Conserved areas are shown shaded. A column is framed, if more than 70% of its residues are similar according to physico-chemical properties.

A BLASTp search of NCBI protein sequence database using as a query the *Ef*Ami1 sequence allowed the construction of a phylogenetic tree (Fig. 1A). The sequences were clustered into three main clades. The first clade contains lytic enzymes originated from *Enterococcus* bacteriophages, the second and the third clades include homologous endolysins from prophages and bacteriophages integrated into the genomes of Gram-positive *Enterococcus* strains and *Enterococcus faecium*, respectively.

Amino acid sequence alignments of close homologue NALAA sequences (> 60% homology) are depicted in Fig. 1B. The alignment allowed the identification of the conserved zinc-ion binding triad (His27, His132, Cys140) based on sequence identity with the amidase-2 domain of LysGH15 from *Staphylococcus* phage G15 (PDB id 4OLS)^42^ (Fig. 1B). The main difference between the phage-derived enzymes with those encoded in the genomes of *E. faecium* is the presence of a 17-mer sequence in the phage enzymes (Fig. 1B). This region is rich in polar amino acid (Thr, Ser) and Gly residues, which is typical for disordered and flexible regions.

### Expression and purification of recombinant *Ef*Ami1

**Figure 2:**
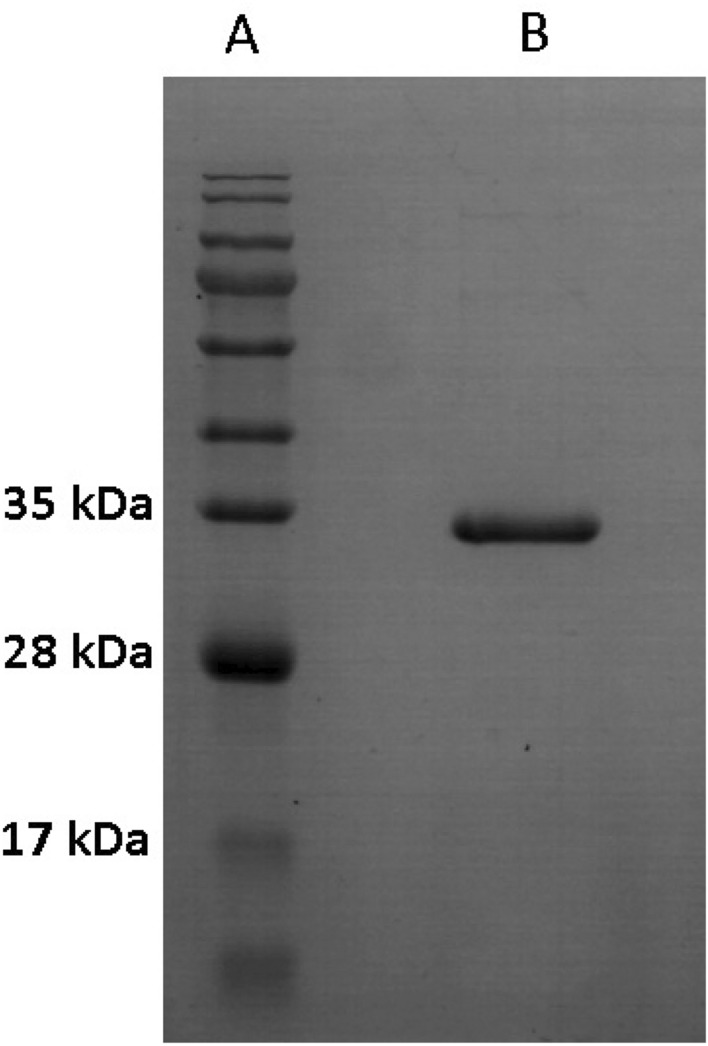
SDS-PAGE analysis of *Ef*Ami1 purity. Protein bands were stained with Coomassie Brilliant Blue R-250. Lane A, Protein Ladder. Lane B, *Ef*Ami1 purified by affinity chromatography on Ni^2+^-IDA-Sepharose.

The full-length gene sequence of *Ef*Ami1 was synthesized and cloned into the T7 expression plasmid pETite. The recombinant plasmid was used for transformation and expression of 6xHis-tagged *Ef*Ami1 in *E. coli* BL21 (DE3) pLysS strain. The extra 6xHis was tagged on the C-terminal of the enzyme, thus enabling *Ef*Ami1 to be rapidly purified as a soluble protein by immobilized metal ion affinity chromatography on a Ni^2+^-iminodiacetic acid (IDA)-Sepharose affinity column (Fig. 2).

### Characterization of the Lytic and bactericidal activity of *Ef*Ami1

#### Effect of pH and Zn^2+^ on lytic activity measured by turbidity reduction assays

**Figure 3:**
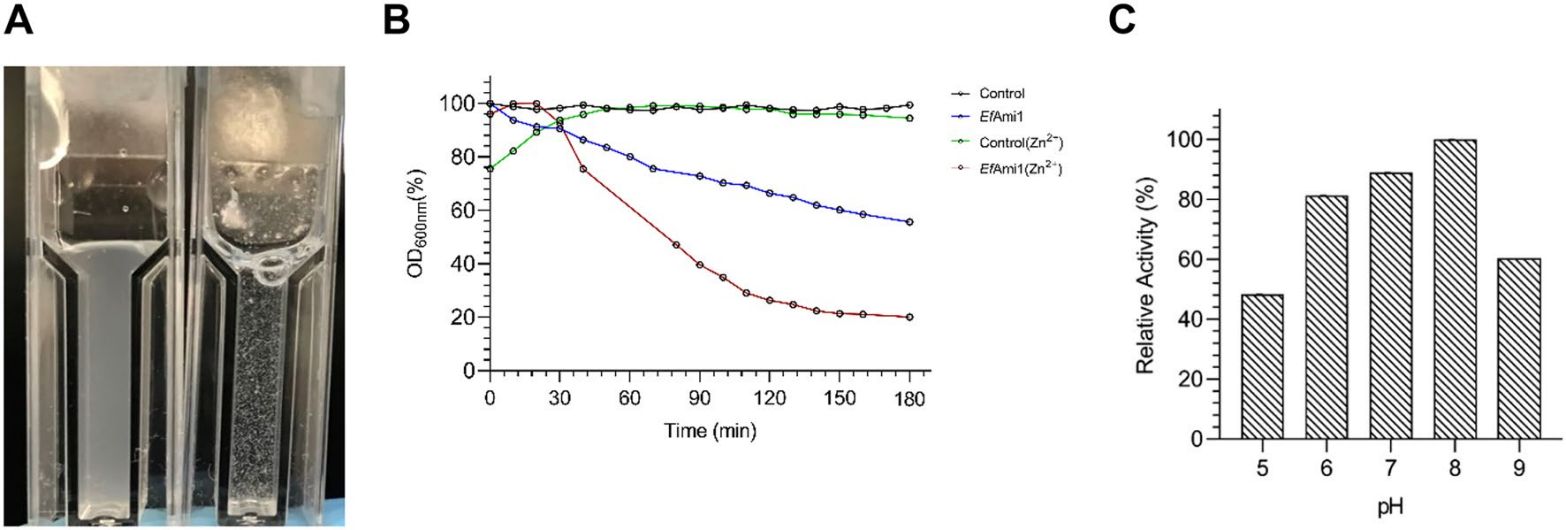
Turbidity reduction assays. (**A**) Visual inspection of the reduction of the turbidity of the *E. faecium* deactivated cells after treatment with recombinant *Ef*Ami1 (100 μg, 2.7 μM) for 120 min at 25 °C. (**B**) Enzyme lytic activity was determined by measuring the reduction of OD_600nm_ using *E. faecium* cells as substrate. *Ef*Ami1 activity was measured in the absence (blue line) or in presence of Zn^2+^ ion (1 mM, red line). Control reactions (without enzyme) in the presence and absence of Zn^2+^ ion (black and green lines, respectively) were also recorded. (**C**) Effect of pH in *Ef*Ami1 activity. All assays were performed using turbidity measurements (OD_600nm_) in each pH value using *E. faecium* cells as substrate.

The effects of pH and Zn^2+^ on the lytic activity of *Ef*Ami1 were determined using *Enterococcus faecium* cells as substrate. Figure 3A shows the reduction in turbidity of *E. faecium* cells after their treatment with recombinant *Ef*Ami1 (100 μg of protein, 2.7 μM) at 25 °C for 180 min (1 mL final volume). The presence of 1 mM Zn^2+^ (Fig. 3B) in the assay mixture significantly enhanced (> 50%) the lytic activity *Ef*Ami1. The lytic activity against *Enterococcus faecium* cells was tested at pH values between 5.0 and 9.0. Figure 3C indicates that *Ef*Ami1 is more active at pH values between 6 and 8, with optimum activity at pH 8.0 (50 mM HEPES/NaOH buffer). The activity was significantly decreased above pH 8.0.

#### Study of the structural stability using differential scanning fluorometry (DSF)

**Figure 4:**
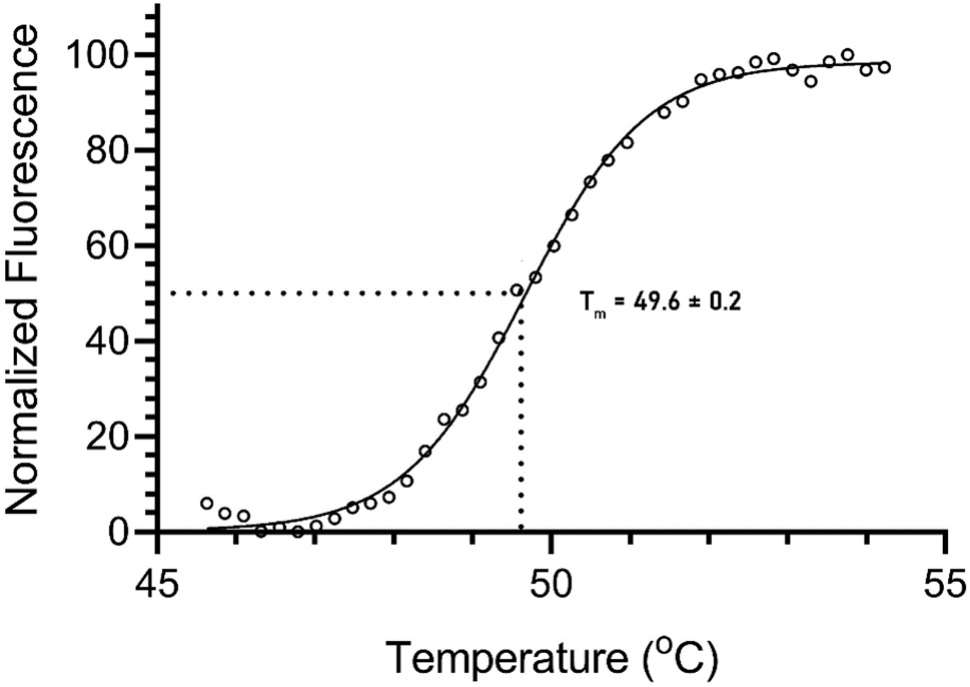
Denaturation curve of purified *Ef*Ami1 using DSF for the determination of its melting temperature at 50 mM HEPES/NaOH buffer, pH 8.

The thermal stability of *Ef*Ami1 was investigated by DSF using the fluorescence dye SYPRO Orange. SYPRO Orange binds *Ef*Ami1 upon denaturation during heat treatment (25–95 °C). Figure 4 shows the denaturation curve of *Ef*Ami1 at optimum activity conditions (i.e., 50 mM HEPES/NaOH buffer, pH 8). A melting temperature (T_m_) of 49.6 ± 0.2 °C was determined. The measured T_m_ for *Ef*Ami1 falls within the expected range for a mesophilic enzyme and is close to that reported for other endolysins^15,17^. However, it is significantly lower to that reported by Żebrowska et al.^43^ for the endolysin from the thermophilic bacteriophage TP-84 (77.6 °C).

#### Lytic specificity of *Ef*Ami1 against Gram-positive and Gram-negative bacteria

**Figure 5:**
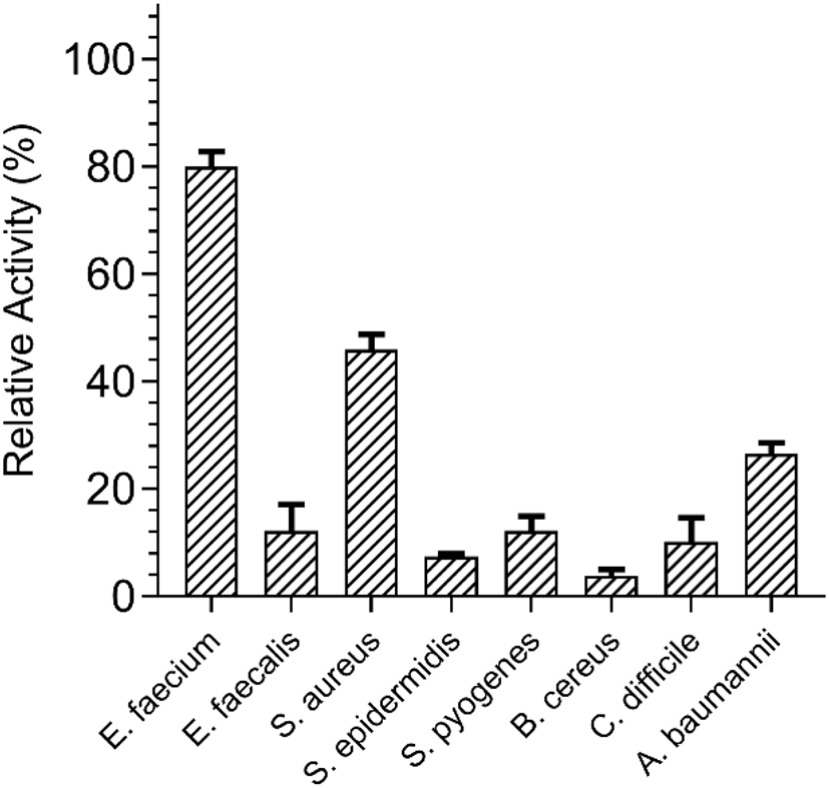
Lytic activity of *Ef*Ami1 against representative Gram-positive and Gram-negative ESKAPE pathogens. All assays were performed using turbidity measurements (OD_600nm_) at pH 8.0. The relative enzyme activity was calculated as a percentage decrease of the initial OD (600 nm) of the cell suspension. In all assays the percentage decrease of the control reaction (in the absence of enzyme) was subtracted.

The lytic activity of *Ef*Ami1 was investigated against a range of Gram-positive (*E. faecium, E. faecalis, S. aureus, S. epidermidis, S. pyogenes, B. cereus, C. difficile*) and Gram-negative (*A. baumannii*) ESKAPE pathogens. The results are shown in Fig. 5. *Ef*Ami1 exhibited lytic activity against all the bacteria tested, although a species-dependent lytic activity was observed. For instance, the enzyme showed its highest lytic activity against *E. faecium* cells. Weaker activity was observed towards *S. aureus, A. baumannii, E. faecalis*, *S. pyogenes* and *C. difficile* cells. This activity spectrum agrees with the results of López-Arvizu et al.^44^. Notably, *Ef*Ami1 exhibits a strain-specific lytic activity and can differentiate between *E. faecium* and *E. faecalis* (Fig. 5)*.* Lytic activity against both Gram-positive and Gram-negative bacteria has also been reported for other endolysins^45^.

#### Evaluation of inhibitory and bactericidal activity of *Ef*Ami1 against live cultures of pathogen bacteria

**Figure 6:**
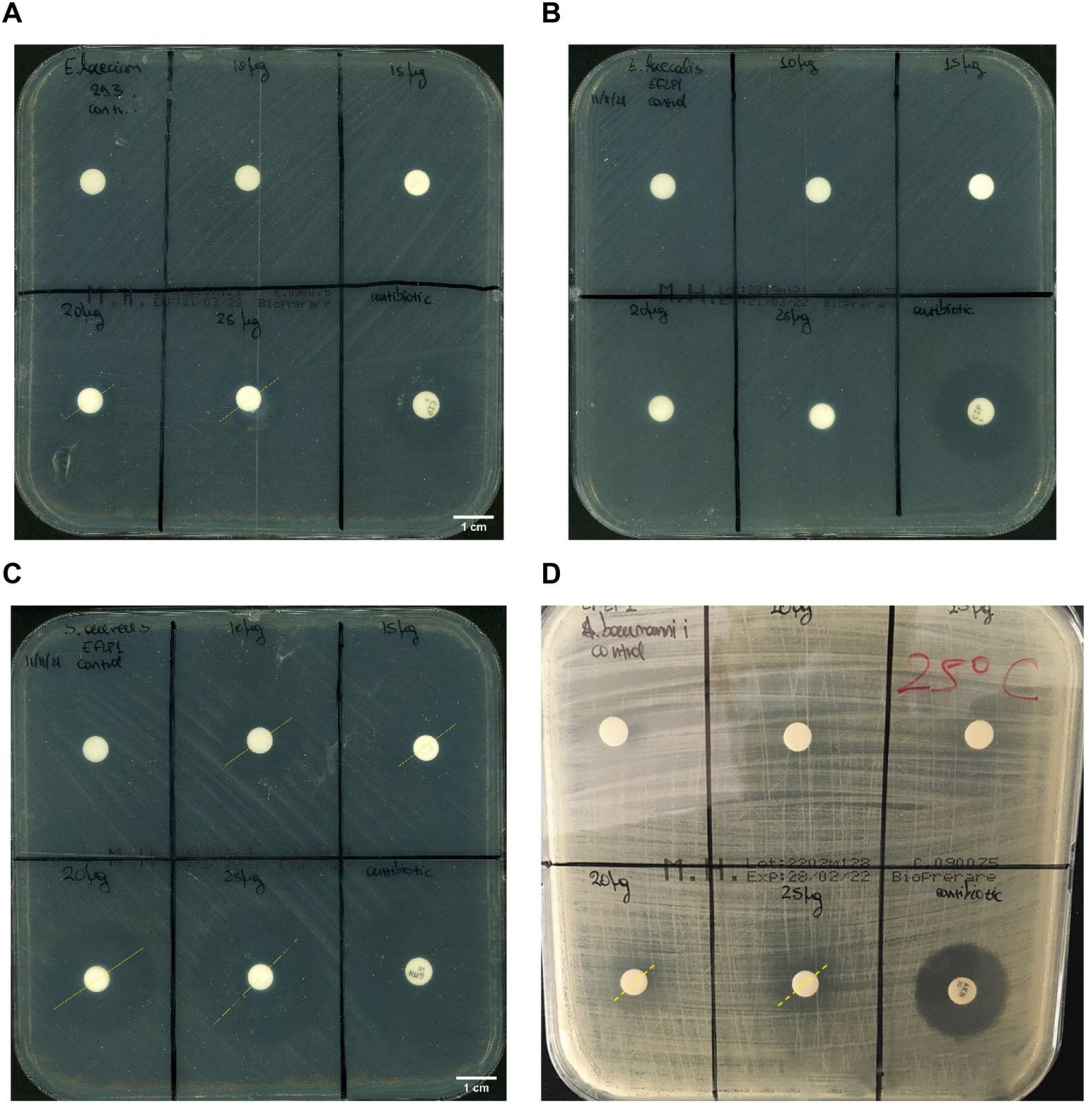
The Kirby–Bauer disk-diffusion method to evaluate the inhibitory and bactericidal activity of different concentration of *Ef*Ami1 against pathogens (*E. faecium, E. faecalis, S. aureus* and *A. baumannii*). (**A**) *Enterococcus faecium*, (**B**) *Enterococcus faecalis*, (**C**) *Staphylococcus aureus*, (**D**) *Acinetobacter baumannii*. Disks contain *Ef*Ami1: 0 μg (control), 10 μg, 15 μg, 20 μg, and 25 μg. The antibiotics used were: Ciprofloxacin (5 μg) for *E. faecium* and *E. faecalis*; gentamicin (30 μg) for *S. aureus*; amikacin (30 μg) for *A. baumannii*.

The Kirby–Bauer disk-diffusion method was carried out to evaluate the inhibitory and bactericidal activity of *Ef*Ami1 against live cultures of selected strains that the turbidity assays (Fig. 6) showed significant lytic activity (*E. faecium, E. faecalis, S. aureus* and *A. baumannii*). The inhibitory and bactericidal activity was evaluated using different amounts (0–25 μg) of purified *Ef*Ami1. Due to completely different biological and chemical conditions that are used in the turbidity assay (Fig. 5) (e.g., dead cells, a solution assay, short incubation time, buffer) and in the disk-diffusion method (live cells, solid phase assay, long incubation time, culture medium), slight discrepancies between them are normally expected. The results (Fig. 6) showed that the presence of enzyme significantly affected the growth of *E. faecium*, creating large inhibition zones at 20 μg (radius of the zone 14 mm) and 25 μg enzyme (18 mm) (Fig. 6A). In contrast, no inhibitory zones were observed in the *E. faecalis* petri dish (Fig. 6B), in agreement with the turbidity assays. Noteworthy activity was also observed against *S. aureus*. In this case, inhibition zones were observed around all tested *Ef*Ami1 concentrations. Zones with radii of 17, 18, 25, and 31 mm were measured using 10, 15, 20, and 25 μg of enzyme, respectively (Fig. 6C). Lower bactericidal activity was observed against *A. baumannii* cells, with 10 mm and 13 mm zones at 20 μg and 25 μg enzyme, respectively (Fig. 6D).

### Structure determination by X-ray crystallography and analysis

**Figure 7:**
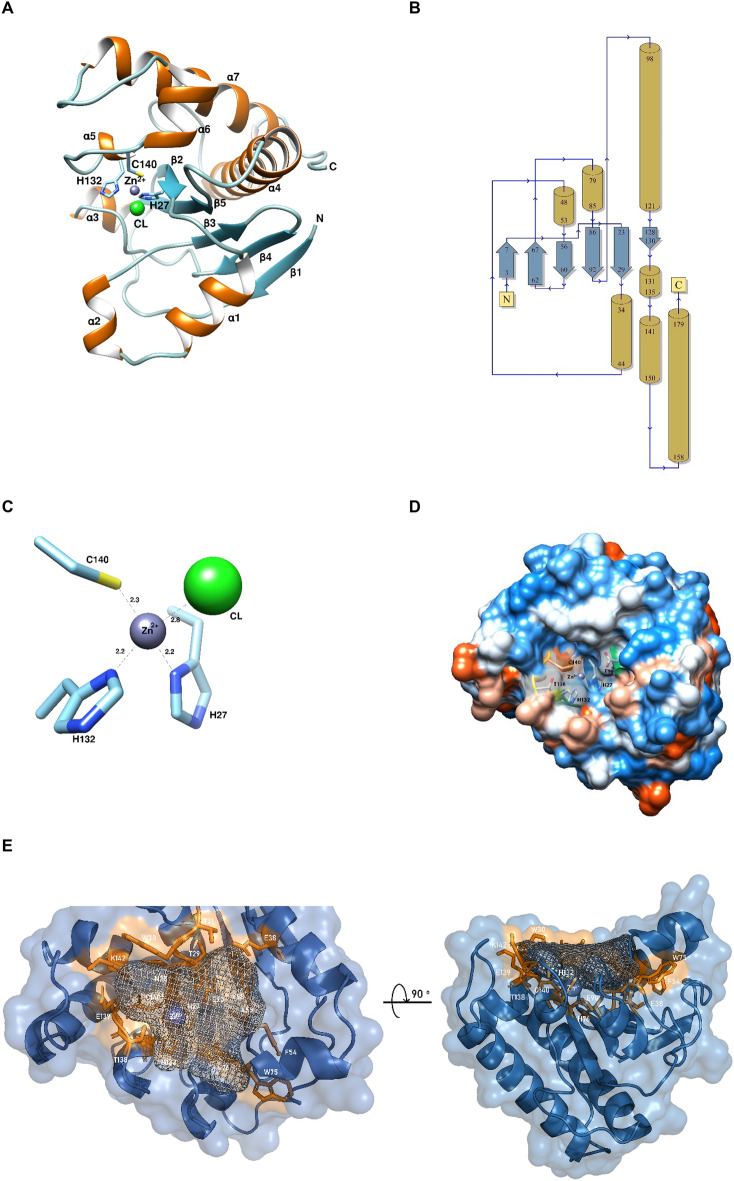
The structure of the amidase-2 domain of *Ef*Ami1 as determined by X-ray crystallography at 1.97 Å resolution. (**A**) Ribbon diagram of the amidase domain of *Ef*Ami1. Secondary structure elements are labelled. The Zn^2+^ and Cl^−^ ions are shown as spheres and labeled. The figure was created using Chimera^46^. (**B**) Topological diagram of the secondary structure elements are illustrated. The figure was created using PDBsum^47^. (**C**) Close-up view of the zinc-binding site. The zinc ligands (His27, His132, Cys140) and distances (in Å) are shown. (**D**) Quantitative hydrophobic representation surface of the amidase-2 domain of *Ef*Ami1*.* Key amino acid residues and the zinc ion are shown and labeled. Color code: blue for the most hydrophilic, to white, to orange red color for the most hydrophobic. The figure was created using UCSF Chimera^46^. (**E**) Mesh representation of the putative peptidoglycan-binding cavity. The zinc ion is shown as a blue sphere. The Python package pyKVFinder was used for cavity detection and characterization. The figure was created using PyMOL^48^.

Purified *Ef*Ami1 was used for crystallization trials using a range of conditions. One condition produced small crystals, which were subjected to diffraction analysis. The results revealed that only the catalytic amidase-2 domain (aa 2–185) was crystallized and its 3D structure was determined at 1.97 Å resolution (Fig. 7A,B, Table 1). The absence of the C-terminal domain from the resolved structure was due to protein degradation, as discussed in another section. Protein degradation was not assessed by mass spectrometry.

**Table 1:** X-ray crystallographic data collection and refinement statistics.

	*Ef*Ami1
*Data collection and processing*	
Beamline	P13 (EMBL-Hamburg)
Wavelength (Å)	0.97620
Resolution (Å)	49.73–1.97 (2.03–1.97)
Space group	*P*4_3_22
Unit cell *a, b, c* (Å)	74.7, 74.7, 133.3
No. of unique reflections	27,661 (2,614)
Completeness (%)	99.9 (99.1)
Multiplicity	25.5 (25.6)
Mosaicity (°)	0.09
*R*_meas_	0.46 (4.63)
CC_1/2_	0.98 (0.42)
Mean (I/σ (I))	9.0 (1.1)
Wilson B factor (Å^2^)	37.3
*Refinement*	
No. of reflections used	27,569
*R*_cryst_/*R*_free_	0.173/0.195
Number of protein residues	184
No. of non-H atoms (protein/solvent)	1,482/190
RMSD in bonds (Å)	0.008
RMSD in angles (º)	0.89
Average B-factor (all/protein/ligands/solvent) (Å^2^)	32.6/30.7/47.1/45.6
Ramachandran favored/outliers (%)	97.8/0.0
Clash score	3.76
PDB id	8C4D

Analysis of the resolved structure revealed the presence of a β-sheet bundle structure, comprising of five β-strands: β1 (amino acids 3–6), β2 (amino acids 23–28), β3 (amino acids 57–60), β4 (amino acids 63–66), β5 (amino acids 86–91). Strands β1, β2 are parallel whilst β3, β4, β5 are anti-parallel compared to the other (Fig. 7A,B). The bundle is framed by four large α-helices (α1, α4, α6, α7) and 3 smaller ones (α2, α3, α5) (Fig. 7A). This secondary structure forms a characteristic cavity in the middle of the structure (Fig. 7D,E). The presence of a zinc ion (Zn^2+^) was observed inside the cavity, although no Zn^2+^ was added to the crystallization buffer (Fig. 7A,C,D,E). The catalytic mechanism of amidases is known to depend on the presence of Zn^2+^, which is located in the peptidoglycan-binding pocket between the binding area of MurNAc and the binding area of the crossed peptide^30,49,50^. The architecture and size of the zinc-binding cavity indicates that it most likely serves as the binding site for the peptidoglycan. The zinc ion in *Ef*Ami1 structure is coordinated by two histidines (His27, His132) and a cysteine residue (Cys140), which are highly conserved (Figs. 1B, 7C,D). The tetrahedral coordination sphere of zinc is completed by a chloride ion.

The algorithm pyKVFider^51^ was used to map the predicted peptidoglycan-binding cavity and the amino acids that are in its vicinity (Fig. 7E). Along with the three conserved amino acids which interact with the zinc ion, the cavity is formed by Asn28, Thr29, Trp30, Thr31, Glu38, Phe54, Ala55, Tyr58, Trp75, His76, Glu90, Thr138, Glu139 and Lys142. The cavity is solvent exposed, which is consistent with its ability to bind a large substrate.

### Structural comparison of *Ef*Ami1 with other amidase-2 endolysins and prediction of the active site residues

**Figure 8:**
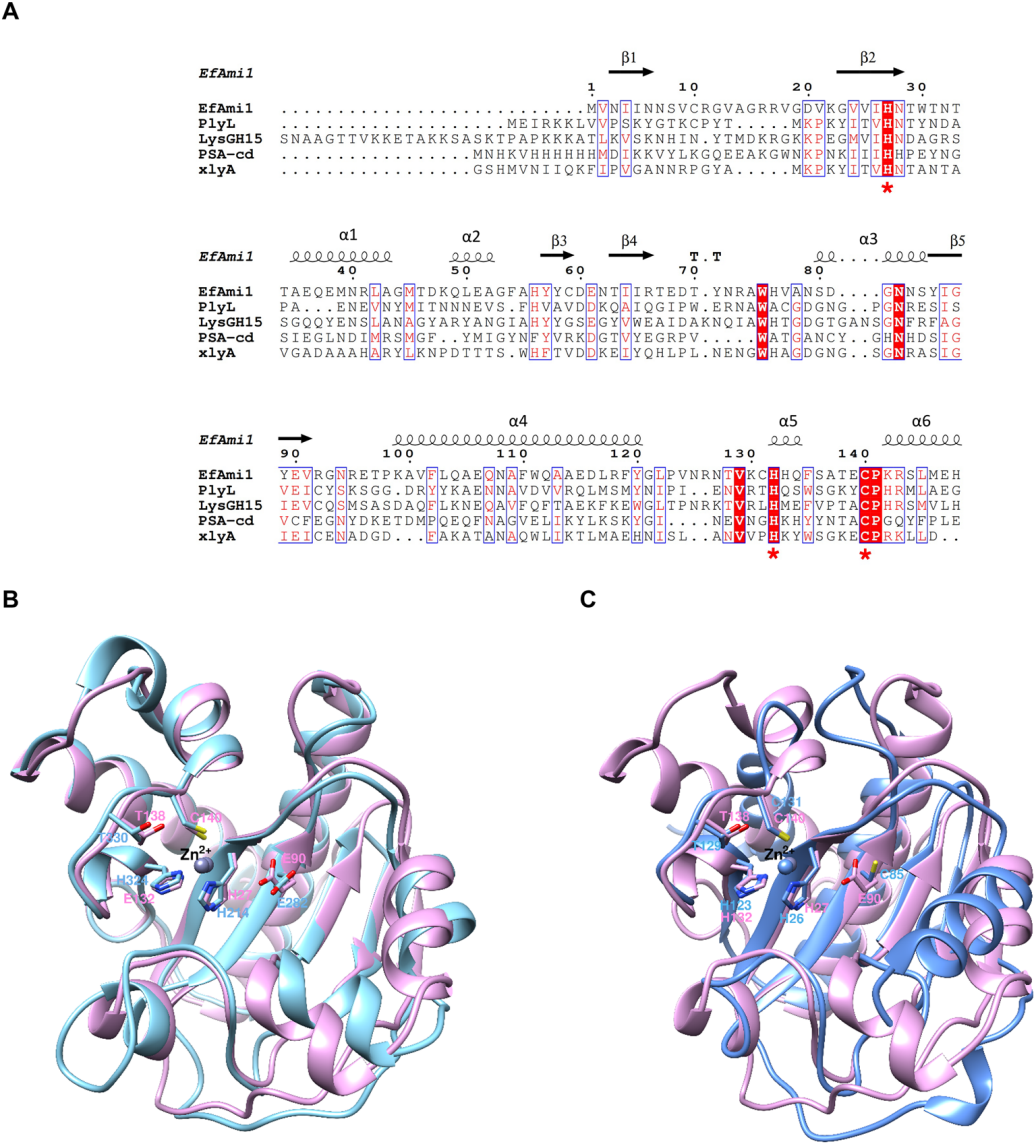
Structural comparison of homologues endolysins. (**A**) Partial structure-based sequence alignment of the amidase domain of five endolysins: *Ef*Ami1; LysGH15 endolysin from Staphylococcus phage G15 (PDB: 4OLS); PlyL prophage endolysin from *Bacillus anthracis* (PDB ID: 1YB0); PSA-cd endolysin from *Clostridium perfringens* (PDB ID: 7F5I); xlyA endolysin from *Bacillus subtilis* (PDB ID: 3HMB). The zinc-binding residues are shown with red stars. *Ef*Ami1 numbering is shown above the alignment. Conserved areas are shown shaded. A column is framed, if more than 70% of its residues are similar according to physico-chemical properties. The figure was created with ESPript^41^. (**B**) Structural superposition of *Ef*Ami1 (pall magenta) onto LysGH15 (greencyan) amidase domain. (**C**) Structural superposition of *Ef*Ami1 (pall magenta) onto PSA-cd (blue) amidase domain. The zinc ions are shown as spheres. The figures were created using UCSF Chimera^46^.

We compared the structure of the *Ef*Ami1 amidase-2 domain with that of other homologues enzymes classified in the NALAA-2 family, such as PlyL (*Bacillus anthracis*)^52^, LysGH15 (*Staphylococcus phage G15*)^42^, PSA-cd (*Clostridium perfringens*)^53^, and xlyA (*Bacillus subtitis*)^54^ (Fig. 8A). All these structures share the same overall fold. The catalytic zinc ion is found in almost the same position in all crystal structures (Fig. 8B,C). Despite similar folds, the amino acid sequences of these proteins share low sequence identity (< 40%) with the *Ef*Ami1 amidase-2 domain. Glu90 in the *Ef*Ami1 structure lies at the identical position of Glu282 in the LysGH15 (rmsd 0.945 Å for 139 atom pairs) (Fig. 8B), Glu90 in PlyL, and Glu93 in xlyA structures (Fig. 8A). Interestingly, in the PSA-cd (rmsd 1.180 Å for 59 atom pairs), there is a Cys residue (Cys85) as structural equivalent of Glu282 (Fig. 8B). Owing to the lack of a Glu residue, PSA-cd uses a Tyr (Tyr51) as the catalytic residue^53^. Based on mutagenesis studies, Glu282 has been proved to play an important role in catalysis^42^. Similarly, Thr138 in the *Ef*Ami1 is located at identical position with that of Thr330 in the LysGH15 (Fig. 8B) and Thr129 in the PSA-cd structure (Fig. 8C). The hydroxyl group of Thr138 probably forms a hydrogen bond with the main chain nitrogen of Cys140 (distance between Thr138-OG1 and Cys140-N is 3.0 Å). This hydrogen bond may contribute to fix the position and orientation of Cys140 as one of the metal-coordinating residues^53^. A similar structural role has been attributed to Thr129 in the PSA-cd^53^. However, Thr330 in LysGH15 contributes to catalytic activity as revealed by the finding that the Thr330Ala mutant demonstrates a 50% decreased activity^42^. Therefore, Glu90 and Thr138 are predicted to be key residues in *Ef*Ami1 (Fig. 8A,B)^42^.

**Figure 9:**
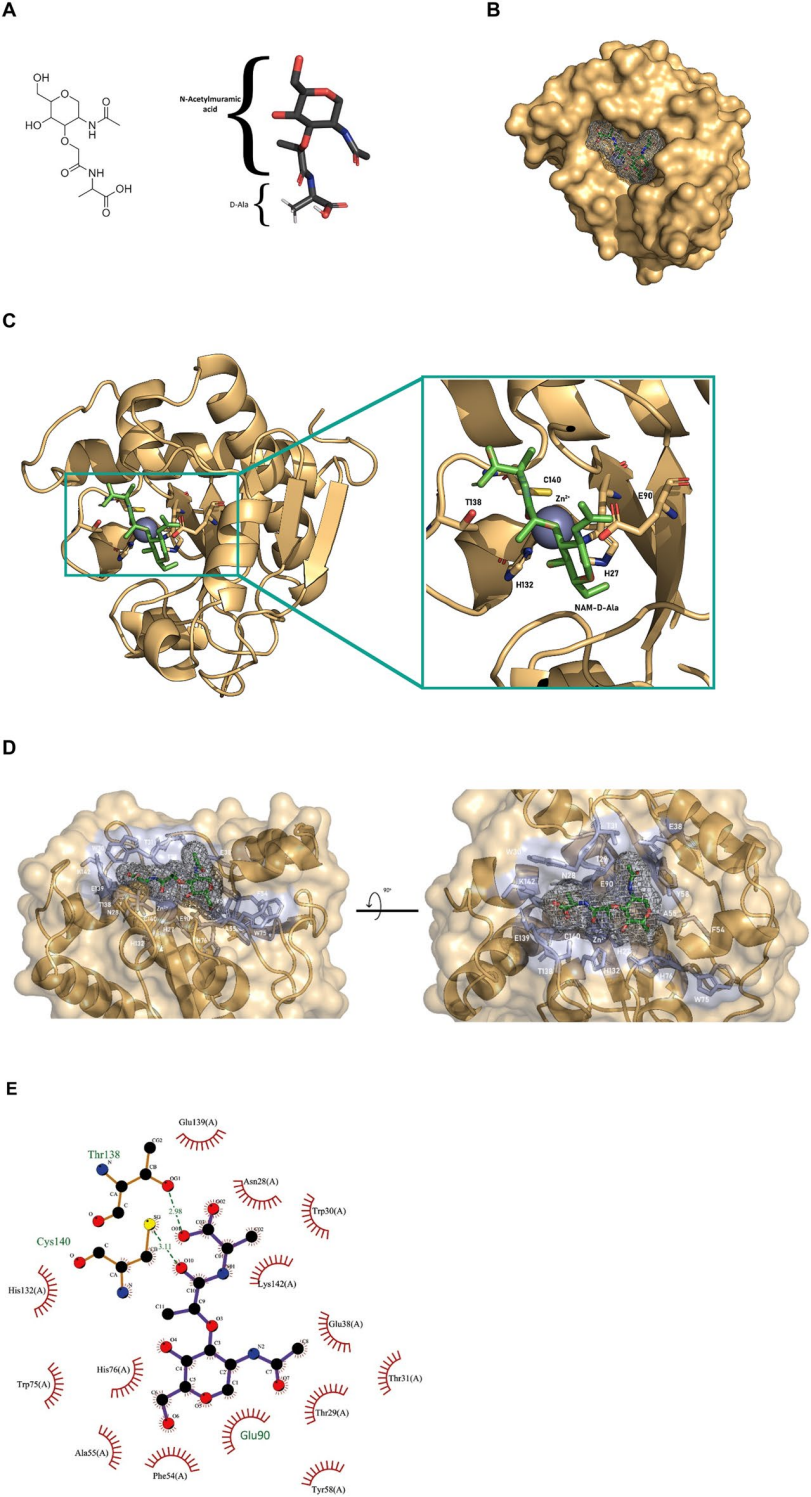
(**A**) The structure of the N-acetylmuramic acid-D-Ala (NAM-D-Ala) molecule used in molecular docking. (**B**) Surface representation of the complex and the positioning of the NAM-D-Ala in the binding site of *Ef*Ami1. (**C**) Ribbon representation of *Ef*Ami1 structure with the NAM-D-Ala bound. The Zn^2+^ ion is shown as a purple sphere. (**D**) A detailed transparent view of the interaction between NAM-D-Ala and the amidase-2 domain (gold). The side chains of selected residues (blue) are shown in stick representation and labelled. The Zn^2+^ ion is shown as a purple sphere. (**E**) 2D representation of the interactions of *Ef*Ami1 amidase-2 structure with the docked NAM-D-Ala molecule using the LigPlot + v.2.2^55^.

The ability of the predicted cavity (Fig. 7E) to bind and interact with the substrate analogue NAM-D-Ala (Fig. 9A) was investigated by molecular docking. As shown in Fig. 9B–E, the ligand NAM-D-Ala binds at the predicted cavity of *Ef*Ami1. The binding energies for the different runs were varied from − 5.59 to − 9.54 kcal/mol. The run with the lowest binding energy (− 9.54 kcal/mol) was selected for further assessment. The inhibition constant (K_i_), which is calculated using the binding energy, was found to be 101.2 nM. The inhibition constant measures the propensity of the complex to dissociate, hence the calculated value justifies the stability of the selected structure. The predicted binding conformation allows the key residues Glu90 and Thr138^42,52^ to interact with NAM-D-Ala. The side chains of Glu90 and Thr138 are oriented towards and interact with the susceptible amide bond of NAM-D-Ala (Fig. 9D,E). In addition, the side chain of Thr138 is in contact with NAM-D-Ala for fixing the substrate in a proper position for the catalytic reaction^53^.

It has been proposed that certain endolysins possess specific regions in their structure that display antimicrobial activity, acting as surfactant-like peptides or providing initial point of association with the bacterial membrane^17,56^. Prediction of regions with antimicrobial activity in *Ef*Ami1 sequence was achieved using the AMPA algorithm^57^. The results of the analysis showed that two regions 8–20 and 292–308 in the amino acid sequence (Fig. 1B) display high antimicrobial activity. Both regions are rich in positively charged residues (Arg and Lys). Inspection of the crystal structure of the amidase-2 domain shows that the region 8–20 is located next to the active site and forms a solvent-exposed flexible loop that connects the β1 and β2 strands (Fig. 7A). Interaction of this region with the membrane can potentially induce perturbing effects on the bilayer structure, making the peptidoglycan more accessible to the active site. The other region (aa 292–308) corresponds to a solvent-exposed loop and a β strand at the C-terminal peptidoglycan-recognition domain (see https://www.alphafold.ebi.ac.uk/entry/A0A7V7GKT0).

### Degradation of *Ef*Ami1

To confirm whether the purified *Ef*Ami1 was degraded during the crystallization experiments and under extended storage at 25 °C, a time course of its structural integrity was assessed using SDS-PAGE analysis. Supplementary Fig. 2A,B shows the degradation profile of the purified *Ef*Ami1 following incubation for 10 days (25 °C) in the presence and absence of Zn^2+^ ion (1 mM) at buffers with different pH values (pH 5.5, 6.5, 7.5). The results showed that acidic conditions (pH 5.5) significantly affect the structural integrity of *Ef*Ami1, causing unspecific degradation. The degradation appears to be independent of the presence or the absence of Zn^2+^ ion (Supplementary Fig. 2A,B). However, as shown in Supplementary Fig. 2C–F, incubation of *Ef*Ami1 at pH 6.5 and 7.5 results in a more specific fragmentation pattern. At pH 7.5, the formation of two large polypeptides with molecular masses of approximately 21 kDa and 16 kDa was observed. The molecular mass of the larger fragment consisted with the size of the amidase-2 domain that was crystallized (amino acids 2–185; according to amino acid sequence the theoretical molecular mass is 21,033 Da) (Fig. 1B). The molecular mass of the smaller fragments is close to that of the C-terminal domain (amino acids 186–324, theoretical molecular mass 15,275 Da). The specific fragmentation of *Ef*Ami1 observed following its prolonged storage is consisted with the results of the x-ray crystallography and the crystallization of the N-terminal domain.

## Conclusions

In this study, a new NALAA from *E. faecium* prophage genome was identified and characterized. In a time of a continuously growing number of available genomes, the exploitation of prophage sequences provides a valuable source of information for the discovery of new endolysin sequences. The main advantage is that prophage sequences are integrated into the bacterial genomes, avoiding the need of phage isolation and purification. *Ef*Ami1 exhibits broad-spectrum activity against both Gram-positive and Gram-negative bacteria. Lytic and antimicrobial assays showed that *Ef*Ami1 displays significant antibacterial activity against *E. faecium*, *S. aureus* and *A. baumannii*. Phylogenetic and structural data from X-ray crystallography revealed that *Ef*Ami1 belongs to the NALAA-2 family. The zinc ion in the active site is coordinated by two histidine residues (His27, His132) and a cysteine residue (Cys140), which are highly conserved. These residues in conjunction with Glu90 and Thr138 are proposed to be essential for enzymatic activity. These results may have broad implications for the design and exploitation of prophage endolysins as new antimicrobial and therapeutic agents.

## Methods

### Bacterial strains

Human clinical samples are not used in the study and only bacterial isolates were used for the current study. The *Enterococcus faecium* strain used in this study was isolated from the Department of Microbiology, “Aghia Sophia” Children’s Hospital, Athens. The antibiogram conducted for this examined strain revealed that it exhibits the following characteristics concerning antibiotic resistance (where: S, susceptible; R, resistant): Ampicillin R, Ciprofloxacin S, Linezolid S, Teicoplanin S, Vancomycin S. *Staphylococcus aureus* ATCC™ 25923 and *Enterococcus faecalis* ATCC™ 29212 were obtained from Microbiologics SCA. *Streprococcus pyogenes*, *Bacillus cereus*, *Staphylococcus epidermidis*, *Acinetobacter baumannii* and *Clostridium difficile* were isolated and obtained as clinical strains from the Department of Microbiology, “Aghia Sophia” Children’s Hospital, Athens.

### Cloning of *Ef*Ami1

The sequence coding for a hypothetical NALAA was identified (Accession No: WP_086274872.1) and was obtained as a synthetic construct (Eurofins Genomics, Germany). PCR was performed to amplify the full-length ORF of the gene and cloned using the In-Fusion HD Cloning Kit (Takara Bio USA, Inc.). The primes used were:

Forward: 5′ GAAGGAGATATACATATGGTGAACATCATTAACAACTCGG 3′.

Reverse: 5′ ATGGTGGTGATGATGCAGAATCAGGTAGATATCGGAAATG 3′.

The PCR reaction was carried out in a total volume of 25 μL, containing: 12.5 μL Clone Amp HiFi PCR Premix, 10 μM forward and reverse primer, 20 ng template DNA and 9.5 μL H_2_O. The conditions used in the thermocycler were an initial denaturation at 98 °C for 4 min. The PCR protocol comprised 35 cycles of 10 s at 95 °C, 15 s at 60 °C (annealing temperature), and 10 s at 72 °C (extension temperature). A final extension time at 72 °C for 10 min was performed after the 35th cycle. The PCR product was run on a 1% (w/v) agarose gel, purified, and ligated to the pETite C-His Vector. The reaction was carried out using the In-Fusion^®^ HD Cloning Kit (Takara Bio USA, Inc.) according to the manufacturer’s instructions. The resulting expression construct (pETite-6His-*Ef*Ami1) was sequenced and used to transform competent *E. coli* BL21(DE3) pLysS cells.

### Heterologous expression of *Ef*Ami1 in *E. coli* BL21 (DE3) pLysS and purification

*E. coli* BL21(DE3) pLysS cells harboring recombinant plasmid were grown at 37 °C in 1 L LB medium containing chloramphenicol (34 μg/mL) and kanamycin (30 mg/mL). The expression of *Ef*Ami1 was induced by the addition of 1 mM isopropyl 1-thio-β-galactopyranoside (IPTG) when the absorbance at 600 nm was approximately 0.6. Cells were harvested by centrifugation (8000×*g*, 20 min) after 4 h. Cell pellet was resuspended in lysis buffer (50 mM NaH_2_PO_4_, 300 mM NaCl, 10 mM imidazole, pH 8), sonicated (50-W, 60 Hz, 5 cycles of 10 s sonication, 30 s interval) in an ice bath (4 °C) and centrifuged twice at 13,000×*g* for 5 min and once at 8000×*g* for 10 min. The supernatant was loaded on a Ni^2+^-IDA-Sepharose (0.5 mL) column previously equilibrated with lysis buffer (50 mM NaH_2_PO_4_, 300 mM NaCl, 10 mM imidazole, pH 8). The adsorbent was washed with twenty column volumes of lysis buffer. The bound 6-His tagged *Ef*Ami1 was eluted with equilibration buffer containing 0.25 M imidazole (2 fractions of 1 mL) and 0.3 M imidazole (1 mL). Fractions were analyzed by SDS-PAGE. All purification steps were performed at 4 °C. Enzyme fractions were pooled, diluted by dropwise addition of glycerol (to 50% v/v final concentration) and stored at − 20 °C. Before use, the enzyme was dialyzed overnight against the appropriate buffer.

### Turbidity reduction assays

Turbidity reduction assays for quantification of enzymatic activity were performed in incubation mixtures (1 mL) in appropriate buffer contained *Ef*Ami1 (100 μg, 2.7 μM) and thermally deactivated *E. faecium* cells (OD_600nm_ of 0.5–0.6). OD_600nm_ was recorded for 180 min at 10 min intervals. Protein concentration was measured using the Bradford method (Bradford, 1976). The effect of Zn^2+^ (1 mM) on enzyme activity was measured using turbidity reduction assays at optimum pH value (pH 8, HEPES/NaOH, 50 mM) with thermally deactivated *E. faecium* cells (OD_600nm_ 0.5–0.6) as substrate.

In order to determine whether *Ef*Ami1 can exert lytic activity against other bacterial species, seven Gram-positive bacteria strains [*E. faecalis* (ATCC: 29212), *E. faecium, S. aureus* (ATCC: 25923), *S. epidermidis*, *S. pyogenes, B. cereus*, and *C. difficile*] and one Gram-negative strain (*A. baumannii*) were used in turbidity assays. In brief, overnight cultures of each bacterial strain were thermally deactivated (121 °C, 30 min) and cells were harvested through centrifugation (8000×*g*). The cells were suspended in 50 mM HEPES/NaOH buffer, pH 8.0 (OD_600nm_ 0.5–0.6) and 100 μg (2.7 μM) purified *Ef*Ami1 were added (1 mL final volume). Reactions were incubated at 25 °C for 180 min and the reduction of OD_600nm_ was recorded every 10 min.

### Effect of pH on *Ef*Ami1 activity

Determination of the optimum pH activity was performed using turbidity reduction assays (1 mL final volume) with the following buffer systems (0.05 M): CH_3_COOH/CH_3_COONa, pH 5.0; MES/NaOH, pH 6.0; HEPES/NaOH, pH 7.0; HEPES/NaOH, pH 8.0; Glycine/NaOH, pH 9. The effect of pH was evaluated using 150 μg (4 μM) purified *Ef*Ami1 and thermally deactivated *Enterococcus faecium* cells as substrate. Protein concentration was measured using the Bradford method^58^. Enzyme relative activity was calculated using the OD_600nm_ of the untreated sample as 100% in each pH value.

### Differential scanning fluorimetry (DSF)

The thermal stability of *Ef*Ami1was measured using DSF on an Applied Biosystems^®^ real-time PCR StepOne™ instrument, as described by Premetis et al.^17^. SYPRO™ Orange dye was used for monitoring the thermal denaturation of the enzyme. Fluorescence monitoring was carried out at 15–95 °C with a rate of 1 °C/min. Assuming a two-state unfolding model, the melting temperature was calculated as the inflection point of the melting curve using the Protein Thermal Shift™ Analysis Software (Applied Biosystems). Assays were performed in triplicate.

### Antimicrobial activity assays using disc diffusion method

Antimicrobial activity assays using disc diffusion method were carried out as described by Premetis et al.^17^. Four bacteria strains were used: *E. faecium, E. faecalis, S. aureus* and *A. baumannii*. The suspension of each strain was adjusted to achieve a turbidity equivalent to 0.5 McFarland turbidity standard. Then, a suspension containing approximately 10^5^ colony-forming units (CFU)/mL for each bacteria strain was prepared. The suspension was swabbed uniformly across Mueller–Hinton (MH) agar plates. Subsequently, filter paper discs (6 mm in diameter) containing different amounts of *Ef*Ami1 [0 μg (control), 10 μg, 15 μg, 20 μg, 25 μg] or a control disk with antibiotic, were placed on the agar surface. The petri dishes were incubated at 25 °C for 24 h.

### Effect of pH on *Ef*Ami1 degradation

The degradation of purified *Ef*Ami1 upon storage at 25 °C was studied at three different buffers over a 10-day period. Samples with purified *Ef*Ami1 were subjected to dialysis against the following buffers: 50 mM CH_3_COOH/CH_3_COONa, pH 5.5; 50 mM MES/NaOH, pH 6.5 and 50 mM HEPES/NaOH, pH 7.5, in the presence and absence of Zn^2+^ ions (1 mM). Protein samples (15 μg) were removed on days 0, 1, 2, 4, 5, 6, 7, and 10 and analyzed by SDS SDS-PAGE.

### Crystallization and data collection

*Ef*Ami1 was concentrated to ~ 11 mg/mL prior to crystallization trials. Thin (< 20 μm) rod-like crystals were produced with the hanging-drop vapor diffusion method at 16 °C using a reservoir solution (0.8 mL) of 20% (w/v) PEG 3000, 0.1 M sodium citrate, pH 5.5 (condition 38 of ShotGun1™ crystallization screen, Molecular Dimensions). Drops consisting of 2 μL of protein solution were mixed with an equal volume of reservoir solution in Linbro crystallization plates. Crystals appeared after approximately 1 week. X-ray diffraction data at 100 K were collected remotely from a single crystal on the P13 beamline at PETRA III (DESY, Hamburg). The crystal was cryoprotected with the inclusion of 20% (v/v) glycerol in the mother liquor prior to flash-freezing in liquid nitrogen.

### Structure determination, refinement, and validation

Initial phases were calculated with molecular replacement. A search in the PDB revealed the amidase-2 domain of LysGH15 (PDB id 4OLS) to have 36% sequence identity with the amidase domain of *Ef*Ami1. No homologues structures were identified for the C-terminal domain. A suitable search model was constructed using SCULPTOR^59^. Molecular replacement was carried out with PHASER^60^ as implemented in PHENIX v. 1.20.1-4487^61^. Inspection of the electron density and the crystal packing suggested the absence of the C-terminal domain in the crystals. The structure was refined to good crystallographic *R* factors (*R*_cryst_ and *R*_free_) and geometry. Validation was carried out with MOLPROBITY^62^, PHENIX, and COOT^63^. X-ray data collection and refinement statistics are shown in Table 1.

### Docking of NAM-D-Ala into the *Ef*Ami1

Prediction of the interactions of the *N*-acetylmuramic acid conjugated with d-Ala (NAM-D-Ala) with *Ef*Ami1 amidase-2 domain were achieved by molecular docking using the AutoDock 4.2.6 and AutoDockTools 1.5.7.^64^. Preparation of the 3D protein structure involved the addition of polar hydrogens and Kolleman charges^65^. The NAM-D-Ala structure was designed using the PyMOL GUI Builder. The Lamarckian genetic algorithm was used for the ligand conformational search^66^. The docking area was defined using AutoGrid, where a 40 Å × 40 Å × 40 Å. 3-D affinity grid was centred on the area of the zinc ion binding site using the means of the three conserved amino acids x, y, z coordinates. The number of runs was 15 with population size 150. The other parameters remained unchanged under these computational conditions.

### Bioinformatics and structure analysis

Sequences homologous to *Ef*Ami1 were sought in the PDB using BLASTp^67^. The resulting sequences were aligned with Clustal Omega^40^. ESPript and ENDscript (http://espript.ibcp.fr) were used for alignment visualization and analysis^68^. Prediction of antimicrobial activity of *Ef*Ami1 regions was accomplished using AMPA^57^. Prediction of the 3D structure of the full protein and the C-terminal domain (amino acids 186–324) was achieved using both AlphaFold^69^ and the I-TASSER server^70^, respectively. Assessment of the putative biological function of the C-terminal domain, was carried out using the COFACTOR^35^ and COACH^36^ servers. COFACTOR deduces protein functions [ligand-binding sites, Enzyme Commission number and Gene Ontology] using structural comparison and protein–protein networks. COACH is a meta-server approach that combines multiple function annotation results (ligand-binding sites) from the COFACTOR, TM-SITE and S-SITE programs^35,36,71^. The python package pyKVFider^51^ was used to map the peptidoglycan-binding cavity and the amino acids that are in its vicinity. The structural analysis was performed using PyMOL^48^, UCSF Chimera^46^, PDBsum web server^47^, and LigPlot + v.2.2^55^.

## Supplementary Information

Supplementary Information.

## Data Availability

The datasets generated and/or analyzed during the current study are available in the Protein Data Bank repository, the accession number for the *Ef*Ami1 crystal structure reported in this paper is 8C4D.

## Abbreviations

AMR: Antimicrobial resistance
CFU: Colony-forming units
DSF: Differential scanning fluorimetry
GH-25: Glycosyl hydrolase family 25
IDA: Iminodiacetic acid
MDR: Multidrug-resistant
MH: Mueller–Hinton
NALAA: *N*-Acetylmuramoyl-l-alanine amidase
NALAA-2: *N*-Acetylmuramoyl-l-alanine amidase-2
NAM-D-Ala: *N*-Acetylmuramic acid-d-Ala
PDE: Peptidoglycan-degrading enzyme
rmsd: Root mean square deviation
WHO: World Health Organization
