# Supplementary material for: Structural and functional features of a broad-spectrum prophage-encoded enzybiotic from Enterococcus faecium
Source: Sci Rep. 2023 May 8;13:7450. doi: 10.1038/s41598-023-34309-2 (PMC10167349; doi:10.1038/s41598-023-34309-2)
Supplement: Supplementary file 1 — Supplementary Information. [file 41598_2023_34309_MOESM1_ESM.docx]

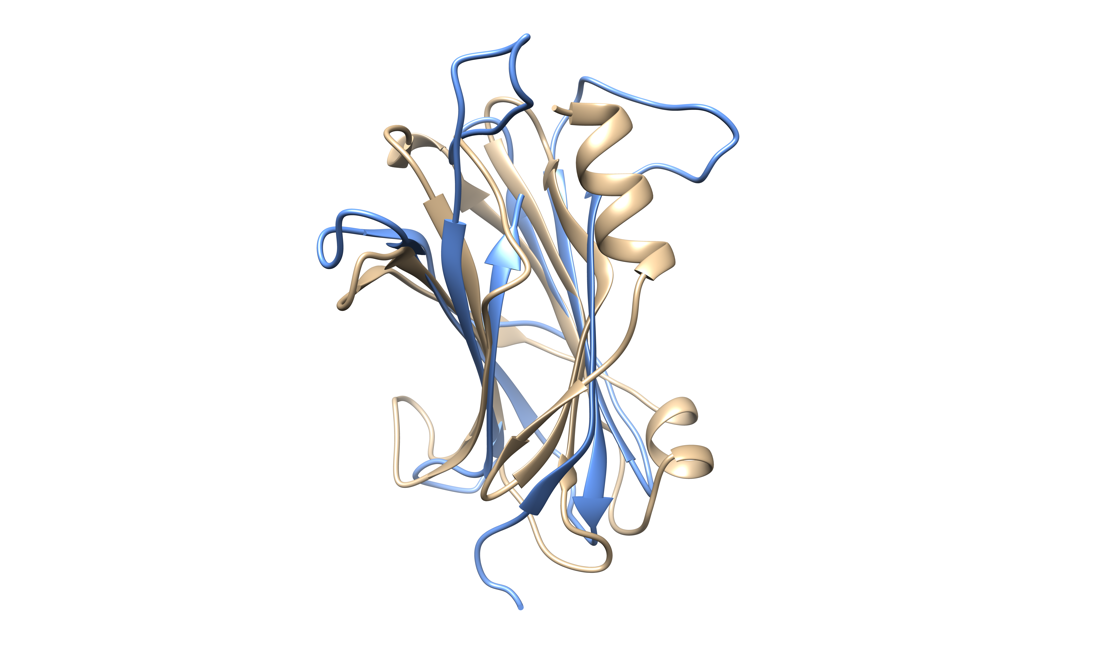


**Supplementary Fig. 1**. Structural comparison of the C-terminal domain of *Ef*Ami1 as predicted by AlphaFold (in coral) with the C-terminal domain (in cornflower blue) of a fungal β-mannosidase from *Trichoderma harzianum.* Structural superposition was carried out with Chimera [46].

| **A**  **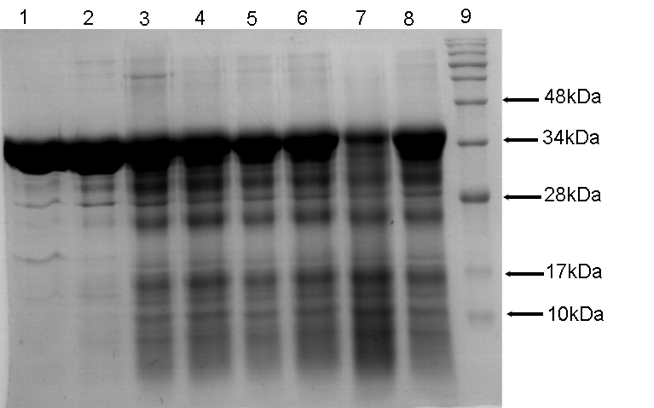** | **B**  **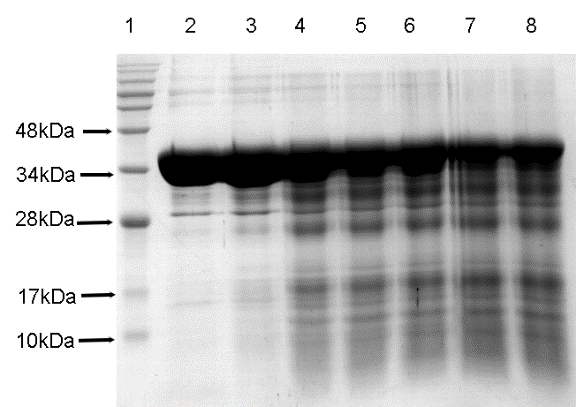** |
| --- | --- |
| **C**  **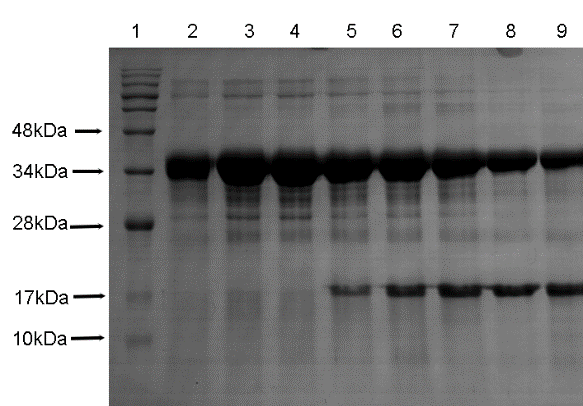** | **D**  **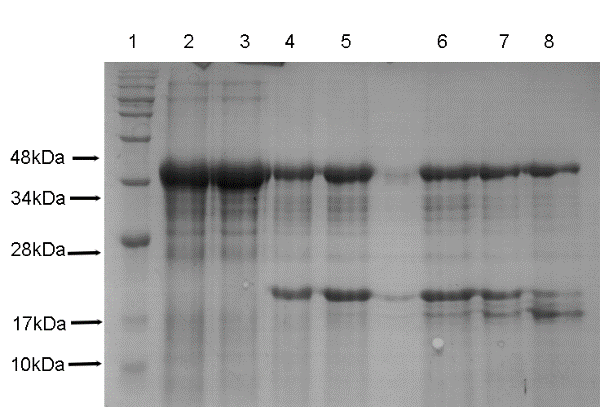** |
| **E**  **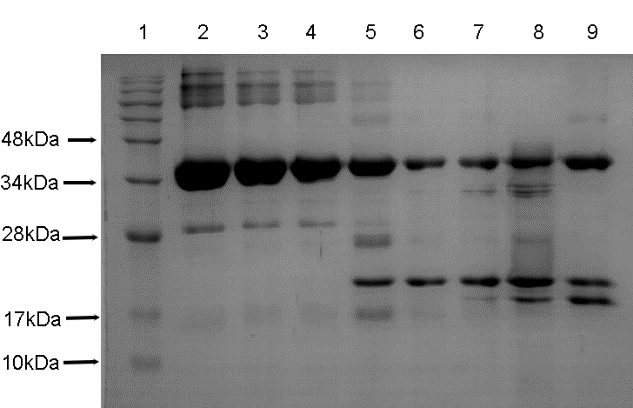** | **F**  **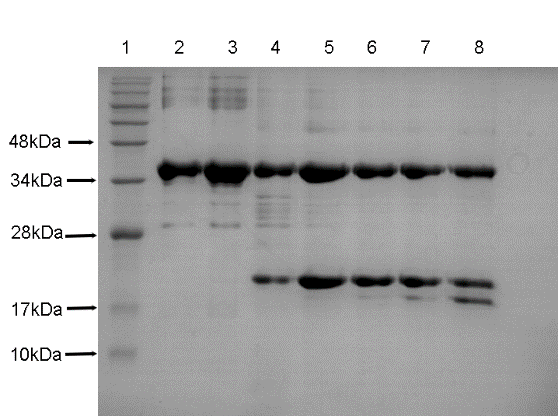** |

**Supplementary Fig. 2.** Effect of pH on *Ef*Ami1 degradation. A, C, E: incubation of *Ef*Ami1 at pH 5.5, 6.5, 7.5, respectively, for 10 days. SDS-PAGE analysis was performed on Day 0, 1, 2, 4, 5, 6, 7 and 10. B, D, F: incubation of *Ef*Ami1 at pH 5.5, 6.5, 7.5, respectively, for 10 days in presence of zinc ion (1 mM). SDS-PAGE analysis was performed on Day 0, 1, 2, 4, 5, 6, 7 and 10. Day 0 corresponds to the day where the enzyme sample was dialyzed at each pH value.
